# Supplementary material for: Estimation of treatment effects following a sequential trial of multiple treatments
Source: Stat Med. 2020 Mar 23;39(11):1593–609. doi: 10.1002/sim.8497 (PMC7217198; doi:10.1002/sim.8497)
Supplement: Supplementary file 1 — Supporting Information [file SIM-39-1593-s001.docx]

**SAS Programs used in SIM**

**Table 3 – RB1 analysis**

**%macro** compute(cas, ist, zst, vst);

/* Legend for input values required to run code. 12 cases will be evaluated to produce results for RB1 estimates of Table 3.

case = case number

ist = Terminal values of the number of interim analyses

zst = Z*

vst = V* */

proc iml;

/* Setting of parameters.

gri = grid size for integral

int = intercept for stopping rule

slope = gradient for stopping rule

ss = number of patients on each treatment arm between interim analyses

deltaz = deltaz for evaluation of equation 5 in paper. */

gri = **100**;

int = **10.93898**;

slope = **0.123134**;

ss = **36**;

deltaz = **0.01**;

cas = &cas;

ist = &ist;

zst = &zst;

vst = &vst;

r = (**1**);

do i = **2** to ist;

r = (r||i);

end;

tscale = (r[**2**] - r[**1**]);

do i = **3** to ist;

tscale = (tscale||r[i] - r[i - **1**]);

end;

v_one = vst/ist;

v = v_one*r;

/* Stopping rule below utilises the double triangular test. */

lb = -int/sqrt(v[**1**]) + **3***slope*v/sqrt(v[**1**]);

ub = int/sqrt(v[**1**]) + slope*v/sqrt(v[**1**]);

width = ub[**1**] - lb[**1**];

ste = width/gri;

lb_one = lb[**1**];

do i = **0** to gri - **1**;

lb[**1**] = lb_one + i*ste;

z_plus = zst + deltaz;

lb[ist] = z_plus/sqrt(v[**1**]);

ub[ist] = z_plus/sqrt(v[**1**]) + **0.000001**;

domain = (lb//ub);

call seq(prob, domain) tscale = tscale;

pl_plus = prob[**1**,];

sumpl_plus = sum(pl_plus);

zst_minus = zst - deltaz;

lb[ist] = zst_minus/sqrt(v[**1**]);

ub[ist] = zst_minus/sqrt(v[**1**]) + **0.000001**;

domain = (lb//ub);

call seq(prob, domain) tscale = tscale;

pl_minus = prob[**1**,];

sumpl_minus = sum(pl_minus);

dif = sumpl_plus - sumpl_minus;

case = (case//cas);

num = (num//i);

istar = (istar//ist);

zstar = (zstar//zst);

vstar = (vstar//vst);

lb1 = (lb1//lb[**1**]);

diff = (diff//dif);

step = (step//ste);

grid = (grid//gri);

v1 = (v1//v_one);

end;

create res1 var{case num istar zstar vstar lb1 diff step grid v1};

append;

quit;

data res2;

set res1;

if istar > **1** then do;

diff0 = max(diff0, diff, **0**);

lb1_0 = min(lb1_0, lb1, **0**);

retain diff0 lb1_0 **0**;

s = diff/diff0;

varg = s*num*step;

sums = sum(sums, s, **0**);

sumvarg = sum(sumvarg, varg, **0**);

retain sums sumvarg **0**;

if num < grid - **1** then delete;

end;

run;

data res3;

set res2;

if istar > **1** then do;

int1 = (sums - **0.5**)*step;

ez1 = (int1 + lb1_0)*sqrt(v1);

thetahat = ez1/v1;

int2 = sumvarg*step;

var1 = (**2***int2 - int1*int1)/v1;

end;

if istar = **1** then do;

grid = **0**;

thetahat = zstar/vstar;

var1 = **0**;

end;

var2 = (**1**/v1) - var1;

se = sqrt(var2);

thetal = thetahat - **1.96***se;

thetau = thetahat + **1.96***se;

keep case istar zstar vstar grid v1 thetahat se thetal thetau;

run;

data results;

set results res3;

run;

**%mend**;

**data** results;

case = **0**;

**run**;

/* 12 cases for which results are required */

%***compute***( **1**, **2**, -**12.0**, **8.160**);

%***compute***( **2**, **3**, -**9.5**, **10.943**);

%***compute***( **3**, **4**, -**8.0**, **12.986**);

%***compute***( **4**, **10**, -**0.5**, **29.833**);

%***compute***( **5**, **8**, **0.0**, **30.359**);

%***compute***( **6**, **13**, **8.0**, **57.337**);

%***compute***( **7**, **9**, **15.0**, **31.819**);

%***compute***( **8**, **6**, **16.0**, **26.963**);

%***compute***( **9**, **6**, **15.5**, **23.745**);

%***compute***(**10**, **5**, **13.5**, **19.744**);

%***compute***(**11**, **5**, **16.0**, **21.600**);

%***compute***(**12**, **3**, **13.5**, **12.527**);

**data** results;

set results;

if case = **0** then delete;

**run**;

**proc** **print** data = results;

**run**;

**Table 3 – RB2 analysis**

options ls = **78** ps = **52** nodate pageno = **1**;

/* The following macro requires 3 input vales;

istar = Terminal values of the number of interim analyses

sestar = number of successes on T1

scstar = number of successes on T2 */

**%macro** setup(istar, sestar, scstar);

data sim;

set gen;

/* Setting of parameters.

int = intercept for stopping rule

slope = gradient for stopping rule

ss = number of patients on each treatment arm between interim analyses

interim = max number of interim analyses

cut = 0 in the case where Z has not exceeding the stopping rule. else cut = 1 and the reverse simulation is discarded. */

int = **10.93898**;

slope = **0.123134**;

ss = **36**;

interim = **25**;

cut = **0**;

array n [**1**:**25**] n1-n25;

array ne [**1**:**25**] ne1-ne25; array nc [**1**:**25**] nc1-nc25;

array se [**1**:**25**] se1-se25; array sc [**1**:**25**] sc1-sc25;

array z [**1**:**25**] z1-z25; array v [**1**:**25**] v1-v25;

array stop [**1**:**25**] stop1-stop25;

istar = &istar;

do j = **1** to istar;

n[j] = **2***j*ss;

ne[j] = j*ss; nc[j] = j*ss;

se[j] = **0**; sc[j] = **0**;

z[j] = **0**; v[j] = **0**;

stop[j] = **0**;

end;

do j = istar + **1** to **25**;

n[j] = **0**;

ne[j] = **0**; nc[j] = **0**;

se[j] = **0**; sc[j] = **0**;

z[j] = **0**; v[j] = **0**;

stop[j] = **0**;

end;

se[istar] = &sestar; sc[istar] = &scstar;

z[istar] = (nc[istar]*se[istar] - ne[istar]*sc[istar])/n[istar];

v[istar] = (ne[istar]*nc[istar]*(se[istar] + sc[istar])*(n[istar] - se[istar] - sc[istar]))/(n[istar]****3**);

stop[istar] = **1**;

**%mend**;

**%macro** ***interim***;

data sim;

set sim;

array n [**1**:**25**] n1-n25;

array ne [**1**:**25**] ne1-ne25; array nc [**1**:**25**] nc1-nc25;

array se [**1**:**25**] se1-se25; array sc [**1**:**25**] sc1-sc25;

array z [**1**:**25**] z1-z25; array v [**1**:**25**] v1-v25;

array stop [**1**:**25**] stop1-stop25;

interim = interim - **1**;

call streaminit(**477568890**);

if interim < istar and cut = **0** then do;

se[interim] = rand('HYPER', ne[interim + **1**], se[interim + **1**], ne[interim]);

sc[interim] = rand('HYPER', nc[interim + **1**], sc[interim + **1**], nc[interim]);

z[interim] = (nc[interim]*se[interim] - ne[interim]*sc[interim])/n[interim];

v[interim] = (ne[interim]*nc[interim]*(se[interim] + sc[interim])*(n[interim] - se[interim] - sc[interim]))/(n[interim]****3**);

stop[interim] = (z[interim] <= -int + **3***slope*v[interim]) + (z[interim] >= int + slope*v[interim]);

cut = stop[interim];

end;

run;

**%mend**;

**%macro** ***results***;

data results;

set sim;

array n [**1**:**25**] n1-n25;

array ne [**1**:**25**] ne1-ne25; array nc [**1**:**25**] nc1-nc25;

array se [**1**:**25**] se1-se25; array sc [**1**:**25**] sc1-sc25;

array z [**1**:**25**] z1-z25; array v [**1**:**25**] v1-v25;

array stop [**1**:**25**] stop1-stop25;

if cut = **1** then delete;

pehat = se[**1**]/ss; pchat = sc[**1**]/ss;

thetahat = z[**1**]/v[**1**];

run;

proc means data = results;

var ss v1 pehat pchat thetahat;

output out = summary mean(v1) = v1 mean(ss) = ss

mean(pehat) = mean_pehat var(pehat) = var_pehat mean(pchat) = mean_pchat var(pchat) = var_pchat

mean(thetahat) = mean_thetahat var(thetahat) = var_thetahat;

run;

/* Our main interest is in thetahat, se_thetahat, thetal and thetau. */

data summary;

set summary;

se_pehat = sqrt(((mean_pehat*(**1** - mean_pehat))/ss) - var_pehat);

pel = mean_pehat - **1.96***se_pehat;

peu = mean_pehat + **1.96***se_pehat;

se_pchat = sqrt(((mean_pchat*(**1** - mean_pchat))/ss) - var_pchat);

pcl = mean_pchat - **1.96***se_pchat;

pcu = mean_pchat + **1.96***se_pchat;

se_thetahat = sqrt((**1**/v1) - var_thetahat);

thetal = mean_thetahat - **1.96***se_thetahat;

thetau = mean_thetahat + **1.96***se_thetahat;

run;

proc print data = summary;

run;

**%mend**;

**%macro** evaluate(istar, sestar, scstar);

%***setup***(&istar, &sestar, &scstar);

%do u = **1** %to **24**;

%***interim***;

%end;

%***results***;

**%mend**;

/* Set number of reverse simulations to be completed - for this analysis and to produce the results in Table 3, 10,000,000 reverse simulations have been used for

each case. */

**data** gen;

do item = **1** to **10000000**;

output;

end;

**run**;

/* Evaluation of 12 cases required for Table 3. */

%***evaluate***(**2**, **35**, **59**);

%***evaluate***(**3**, **68**, **87**);

%***evaluate***(**4**, **102**, **118**);

%***evaluate***(**10**, **284**, **285**);

%***evaluate***(**8**, **201**, **201**);

%***evaluate***(**13**, **275**, **259**);

%***evaluate***(**9**, **252**, **222**);

%***evaluate***(**6**, **120**, **88**);

%***evaluate***(**6**, **161**, **130**);

%***evaluate***(**5**, **135**, **108**);

%***evaluate***(**5**, **124**, **92**);

%***evaluate***(**3**, **82**, **55**);

**Table 7 – RB2 analysis from 4^th^ interim**

/* Adjust the location of the SAS library to personal specification */

libname perm 'G:\Home\Research\REALISE-Uganda\Estimation';

/* The generation of results for Table 7 require 3 separate reverse simulations. See Section 5 of the paper for further details. This file is used to produce estimates for

theta12, theta23 and theta24. */

**%macro** ***interim***;

data sim;

set sim;

array n[**16**, **4**];

array s[**16**, **4**];

array z[**24**, **4**];

array v[**24**, **4**];

array invarone[**24**];

array zsum[**6**, **4**];

array vsum[**6**, **4**];

array varonesum[**6**];

array c[**6**, **4**];

array sinput[**16**];

array lastint[**4**];

array treat[**16**];

array conc[**6**];

interim = interim - **1**;

seed = **477568890** + interim;

call streaminit(seed);

do st = **1** to **16**;

if interim < lastint[treat[st]] and s[st, interim + **1**] > **0** then do;

s[st, interim] = rand('HYPER', n[st, interim + **1**], s[st, interim + **1**], n[st, interim]);

end;

end;

do site = **1** to **4**;

do tra = **1** to **3**;

do trb = tra + **1** to **4**;

comp = **6***(site - **1**) + (tra = **1**)*(trb - **1**) + (tra = **2**)*(trb + **1**) + (tra = **3**)*(trb + **2**);

sta = site + **4***(tra - **1**);

stb = site + **4***(trb - **1**);

if interim <= lastint[tra] and interim <= lastint[trb] then do;

z[comp, interim] = ((n[stb, interim]*s[sta, interim] - n[sta, interim]*s[stb, interim])/(n[sta, interim] + n[stb, interim]));

v[comp, interim] = ((n[sta, interim]*n[stb, interim]*(s[sta, interim] + s[stb, interim])

*(n[sta, interim] + n[stb, interim] - s[sta, interim] - s[stb, interim])/((n[sta, interim]+ n[stb, interim])****3**)));

end;

if interim = **1** then do;

invarone[comp] = ((n[sta, **1**] + n[stb, **1**])*v[comp, **1**])/(n[sta, **1**] + n[stb, **1**] - **1**);

/**************************************************************

The (-1) in the above line is explained in section 5 of the paper, specifically in equation 7.

**************************************************************/

end;

end;

end;

end;

stillin = **0**;

equiv = **0**;

do tra = **1** to **3**;

do trb = tra + **1** to **4**;

komp = (tra = **1**)*(trb - **1**) + (tra = **2**)*(trb + **1**) + (tra = **3**)*(trb + **2**);

zsum[komp, interim] = z[komp, interim] + z[komp + **6**, interim] + z[komp + **12**, interim] + z[komp + **18**, interim];

vsum[komp, interim] = v[komp, interim] + v[komp + **6**, interim] + v[komp + **12**, interim] + v[komp + **18**, interim];

if interim = **1** then do;

varonesum[komp] = **1**/(invarone[komp] + invarone[komp + **6**] + invarone[komp + **12**] + invarone[komp + **18**]);

/**************************************************************

The (-1) in the above line is explained in section 5 of the paper, specifically in equation 7.

**************************************************************/

end;

b1 = -int - slope*vsum[komp, interim];

b2 = int - **3***slope*vsum[komp, interim];

if (b2 > **0**) then b2 = **0**;

b3 = -b2;

b4 = -b1;

if (b2 > **0**) then b2 = **0.0001**;

if (b3 < **0**) then b3 = **0.0001**;

c[komp, interim] = ((**1** + (zsum[komp, interim] > b1) + (zsum[komp, interim] >= b2) + (zsum[komp, interim] > b3) + (zsum[komp, interim] >= b4)));

if interim = lastint[tra] and interim = lastint[trb] then do;

stillin = stillin + **1**;

end;

if interim = lastint[tra] and interim < lastint[trb] then do;

complete = min(complete, (**1** - (c[komp, interim] = **5**)));

complete = min(complete, (**1** - (c[komp, interim] > **1**)*(conc[komp] = **1**)));

complete = min(complete, (**1** - (c[komp, interim] = **1**)*(conc[komp] > **1**)));

stillin = stillin + **1**;

equiv = equiv + (c[komp, interim] = **3**);

end;

if interim < lastint[tra] and interim = lastint[trb] then do;

complete = min(complete, (**1** - (c[komp, interim] = **1**)));

complete = min(complete, (**1** - (c[komp, interim] < **5**)*(conc[komp] = **5**)));

complete = min(complete, (**1** - (c[komp, interim] = **5**)*(conc[komp] < **5**)));

stillin = stillin + **1**;

equiv = equiv + (c[komp, interim] = **3**);

end;

if interim < lastint[tra] and interim < lastint[trb] then do;

complete = min(complete, **1** - (c[komp, interim] = **1**) - (c[komp, interim] = **5**));

stillin = stillin + **1**;

equiv = equiv + (c[komp, interim] = **3**);

end;

if interim <= lastint[tra] and interim <= lastint[trb] then do;

complete = min(complete, **1** - (equiv = stillin));

end;

end;

end;

run;

proc means data = sim;

var interim;

run;

**%mend**;

**%macro** ***results***;

data results;

set sim;

array z[**24**, **4**];

array v[**24**, **4**];

array zsum[**6**, **4**];

array vsum[**6**, **4**];

array thetahat[**6**];

array varonesum[**6**];

array c[**6**, **4**];

array sinput[**16**];

array lastint[**4**];

array treat[**16**];

array conc[**6**];

if complete = **0** then delete;

do comp = **1** to **6**;

thetahat[comp] = zsum[comp, **1**]*varonesum[comp];

end;

run;

proc means data = results noprint;

var thetahat1 thetahat2 thetahat3 thetahat4 thetahat5 thetahat6 varonesum1 varonesum2 varonesum3 varonesum4 varonesum5 varonesum6;

output out = summary mean(thetahat1) = th12 mean(thetahat2) = th13 mean(thetahat3) = th14

mean(thetahat4) = th23 mean(thetahat5) = th24 mean(thetahat6) = th34

var(thetahat1) = var_th12 var(thetahat2) = var_th13 var(thetahat3) = var_th14

var(thetahat4) = var_th23 var(thetahat5) = var_th24 var(thetahat6) = var_th34

mean(varonesum1) = vee12 mean(varonesum2) = vee13 mean(varonesum3) = vee14

mean(varonesum4) = vee23 mean(varonesum5) = vee24 mean(varonesum6) = vee34;

run;

data summary;

set summary;

array th[**6**] th12 th13 th14 th23 th24 th34;

array var_th[**6**] var_th12 var_th13 var_th14 var_th23 var_th24 var_th34;

array se_th[**6**] se_th12 se_th13 se_th14 se_th23 se_th24 se_th34;

array vee[**6**] vee12 vee13 vee14 vee23 vee24 vee34;

array thl[**6**] thl12 thl13 thl14 thl23 thl24 thl34;

array thu[**6**] thu12 thu13 thu14 thu23 thu24 thu34;

do comp = **1** to **6**;

se_th[comp] = sqrt(vee[comp] - var_th[comp]);

thl[comp] = th[comp] - **1.96***se_th[comp];

thu[comp] = th[comp] + **1.96***se_th[comp];

end;

proc print data = summary;

run;

**%mend**;

**%macro** ***evaluate***;

%do u = **1** %to **4**;

%***interim***;

%end;

%***results***;

**%mend**;

**data** input;

array n[**16**, **4**];

array sinput[**16**];

array lastint[**4**];

array conc[**6**];

input n1-n64 sinput1-sinput16 lastint1-lastint4 conc1-conc6;

cards;

11 18 30 41

10 16 25 33

7 17 25 35

8 21 28 35

12 24 31 39

6 13 25 30

7 16 22 35

11 19 30 40

9 19 29 39

7 15 24 32

9 17 25 32

11 21 30 41

9 15 23 36

9 20 32 42

11 19 28 32

7 18 25 34

35 25 20 20 25 13 21 11 27 22 13 15 24 24 14 13

4 4 4 4

5 0 0 0 0 0

;

**run**;

**data** sim;

set input;

array n[**16**, **4**];

array s[**16**, **4**];

array z[**24**, **4**];

array v[**24**, **4**];

array zsum[**6**, **4**];

array vsum[**6**, **4**];

array c[**6**, **4**];

array sinput[**16**];

array lastint[**4**];

array treat[**16**];

array conc[**6**];

int = **10.90266**;

slope = **0.12380**;

interim = **5**;

complete = **1**;

do i = **1** to **4**;

do st = **1** to **16**;

s[st, i] = **0**;

end;

end;

do i = **1** to **4**;

do comp = **1** to **24**;

z[comp, i] = **0**;

v[comp, i] = **0**;

end;

end;

do i = **1** to **4**;

do komp = **1** to **6**;

zsum[komp, i] = **0**;

vsum[komp, i] = **0**;

c[komp, i] = **0**;

end;

end;

do st = **1** to **16**;

treat[st] = int((st + **3**)/**4**);

s[st, lastint[treat[st]]] = sinput[st];

end;

/*******************************************************************

Here is where the number of reverse simulations is set. We have used 10,000,000 for Table 7.

*******************************************************************/

do rep = **1** to **10000000**;

output;

end;

**run**;

%***evaluate***;

**Table 7 – RB2 analysis from 5^th^ interim**

/* Adjust the location of the SAS library to personal specification */

libname perm 'G:\Home\Research\REALISE-Uganda\Estimation';

/* The generation of results for Table 7 require 3 separate reverse simulations. See Section 5 of the paper for further details. This file is used to produce estimates for

theta14 and theta34. */

**%macro** ***interim***;

data sim;

set sim;

array n[**16**, **5**];

array s[**16**, **5**];

array z[**24**, **5**];

array v[**24**, **5**];

array invarone[**24**];

array zsum[**6**, **5**];

array vsum[**6**, **5**];

array varonesum[**6**];

array c[**6**, **5**];

array sinput[**16**];

array lastint[**4**];

array treat[**16**];

array conc[**6**];

interim = interim - **1**;

seed = **477568890** + interim;

call streaminit(seed);

do st = **1** to **16**;

if interim < lastint[treat[st]] and s[st, interim + **1**] > **0** then do;

s[st, interim] = rand('HYPER', n[st, interim + **1**], s[st, interim + **1**], n[st, interim]);

end;

end;

do site = **1** to **4**;

do tra = **1** to **3**;

do trb = tra + **1** to **4**;

comp = **6***(site - **1**) + (tra = **1**)*(trb - **1**) + (tra = **2**)*(trb + **1**) + (tra = **3**)*(trb + **2**);

sta = site + **4***(tra - **1**);

stb = site + **4***(trb - **1**);

if interim <= lastint[tra] and interim <= lastint[trb] then do;

z[comp, interim] = ((n[stb, interim]*s[sta, interim] - n[sta, interim]*s[stb, interim])/(n[sta, interim] + n[stb, interim]));

v[comp, interim] = ((n[sta, interim]*n[stb, interim]*(s[sta, interim] + s[stb, interim])

*(n[sta, interim] + n[stb, interim] - s[sta, interim] - s[stb, interim])/((n[sta, interim]+ n[stb, interim])****3**)));

end;

if interim = **1** then do;

invarone[comp] = ((n[sta, **1**] + n[stb, **1**])*v[comp, **1**])/(n[sta, **1**] + n[stb, **1**] - **1**);

/**************************************************************

The (-1) in the above line is explained in section 5 of the paper, specifically in equation 7.

**************************************************************/

end;

end;

end;

end;

stillin = **0**;

equiv = **0**;

do tra = **1** to **3**;

do trb = tra + **1** to **4**;

komp = (tra = **1**)*(trb - **1**) + (tra = **2**)*(trb + **1**) + (tra = **3**)*(trb + **2**);

zsum[komp, interim] = z[komp, interim] + z[komp + **6**, interim] + z[komp + **12**, interim] + z[komp + **18**, interim];

vsum[komp, interim] = v[komp, interim] + v[komp + **6**, interim] + v[komp + **12**, interim] + v[komp + **18**, interim];

if interim = **1** then do;

varonesum[komp] = **1**/(invarone[komp] + invarone[komp + **6**] + invarone[komp + **12**] + invarone[komp + **18**]);

/**************************************************************

The (-1) in the above line is explained in section 5 of the paper, specifically in equation 7.

**************************************************************/

end;

b1 = -int - slope*vsum[komp, interim];

b2 = int - **3***slope*vsum[komp, interim];

if (b2 > **0**) then b2 = **0**;

b3 = -b2;

b4 = -b1;

if (b2 > **0**) then b2 = **0.0001**;

if (b3 < **0**) then b3 = **0.0001**;

c[komp, interim] = ((**1** + (zsum[komp, interim] > b1) + (zsum[komp, interim] >= b2) + (zsum[komp, interim] > b3) + (zsum[komp, interim] >= b4)));

if interim = lastint[tra] and interim = lastint[trb] then do;

stillin = stillin + **1**;

end;

if interim = lastint[tra] and interim < lastint[trb] then do;

complete = min(complete, (**1** - (c[komp, interim] = **5**)));

complete = min(complete, (**1** - (c[komp, interim] > **1**)*(conc[komp] = **1**)));

complete = min(complete, (**1** - (c[komp, interim] = **1**)*(conc[komp] > **1**)));

stillin = stillin + **1**;

equiv = equiv + (c[komp, interim] = **3**);

end;

if interim < lastint[tra] and interim = lastint[trb] then do;

complete = min(complete, (**1** - (c[komp, interim] = **1**)));

complete = min(complete, (**1** - (c[komp, interim] < **5**)*(conc[komp] = **5**)));

complete = min(complete, (**1** - (c[komp, interim] = **5**)*(conc[komp] < **5**)));

stillin = stillin + **1**;

equiv = equiv + (c[komp, interim] = **3**);

end;

if interim < lastint[tra] and interim < lastint[trb] then do;

complete = min(complete, **1** - (c[komp, interim] = **1**) - (c[komp, interim] = **5**));

stillin = stillin + **1**;

equiv = equiv + (c[komp, interim] = **3**);

end;

if interim <= lastint[tra] and interim <= lastint[trb] then do;

complete = min(complete, **1** - (equiv = stillin));

end;

end;

end;

run;

proc means data = sim;

var interim;

run;

**%mend**;

**%macro** ***results***;

data results;

set sim;

array z[**24**, **5**];

array v[**24**, **5**];

array zsum[**6**, **5**];

array vsum[**6**, **5**];

array thetahat[**6**];

array varonesum[**6**];

array c[**6**, **12**];

array sinput[**16**];

array lastint[**4**];

array treat[**16**];

array conc[**6**];

if complete = **0** then delete;

do comp = **1** to **6**;

thetahat[comp] = zsum[comp, **1**]*varonesum[comp];

end;

run;

proc means data = results noprint;

var thetahat1 thetahat2 thetahat3 thetahat4 thetahat5 thetahat6 varonesum1 varonesum2 varonesum3 varonesum4 varonesum5 varonesum6;

output out = summary mean(thetahat1) = th12 mean(thetahat2) = th13 mean(thetahat3) = th14

mean(thetahat4) = th23 mean(thetahat5) = th24 mean(thetahat6) = th34

var(thetahat1) = var_th12 var(thetahat2) = var_th13 var(thetahat3) = var_th14

var(thetahat4) = var_th23 var(thetahat5) = var_th24 var(thetahat6) = var_th34

mean(varonesum1) = vee12 mean(varonesum2) = vee13 mean(varonesum3) = vee14

mean(varonesum4) = vee23 mean(varonesum5) = vee24 mean(varonesum6) = vee34;

run;

data summary;

set summary;

array th[**6**] th12 th13 th14 th23 th24 th34;

array var_th[**6**] var_th12 var_th13 var_th14 var_th23 var_th24 var_th34;

array se_th[**6**] se_th12 se_th13 se_th14 se_th23 se_th24 se_th34;

array vee[**6**] vee12 vee13 vee14 vee23 vee24 vee34;

array thl[**6**] thl12 thl13 thl14 thl23 thl24 thl34;

array thu[**6**] thu12 thu13 thu14 thu23 thu24 thu34;

do comp = **1** to **6**;

se_th[comp] = sqrt(vee[comp] - var_th[comp]);

thl[comp] = th[comp] - **1.96***se_th[comp];

thu[comp] = th[comp] + **1.96***se_th[comp];

end;

run;

proc print data = summary;

run;

**%mend**;

**%macro** ***evaluate***;

%do u = **1** %to **5**;

%***interim***;

%end;

%***results***;

**%mend**;

**data** input;

array n[**16**, **5**];

array sinput[**16**];

array lastint[**4**];

array conc[**6**];

input n1-n80 sinput1-sinput16 lastint1-lastint4 conc1-conc6;

cards;

11 18 30 41 50

10 16 25 33 41

7 17 25 35 44

8 21 28 35 45

12 24 31 39 0

6 13 25 30 0

7 16 22 35 0

11 19 30 40 0

9 19 29 39 48

7 15 24 32 40

9 17 25 32 42

11 21 30 41 50

9 15 23 36 50

9 20 32 42 47

11 19 28 32 40

7 18 25 34 43

41 30 26 27 25 13 21 11 33 28 21 18 32 27 18 16

5 4 5 5

5 0 5 0 0 0

;

**run**;

**data** sim;

set input;

array n[**16**, **5**];

array s[**16**, **5**];

array z[**24**, **5**];

array v[**24**, **5**];

array zsum[**6**, **5**];

array vsum[**6**, **5**];

array c[**6**, **5**];

array sinput[**16**];

array lastint[**4**];

array treat[**16**];

array conc[**6**];

int = **10.90266**;

slope = **0.12380**;

interim = **6**;

complete = **1**;

do i = **1** to **5**;

do st = **1** to **16**;

s[st, i] = **0**;

end;

end;

do i = **1** to **5**;

do comp = **1** to **24**;

z[comp, i] = **0**;

v[comp, i] = **0**;

end;

end;

do i = **1** to **5**;

do komp = **1** to **6**;

zsum[komp, i] = **0**;

vsum[komp, i] = **0**;

c[komp, i] = **0**;

end;

end;

do st = **1** to **16**;

treat[st] = int((st + **3**)/**4**);

s[st, lastint[treat[st]]] = sinput[st];

end;

/*******************************************************************

Here is where the number of reverse simulations is set. We have used 10,000,000 for Table 7.

*******************************************************************/

do rep = **1** to **10000000**;

output;

end;

**run**;

%***evaluate***;

**data** perm.batch5_1;

set results;

keep thetahat1 thetahat2 thetahat3 thetahat4 thetahat5 thetahat6 varonesum1 varonesum2 varonesum3 varonesum4 varonesum5 varonesum6;

**run**;

**Table 7 – RB2 analysis from 12^th^ interim**

/* Adjust the location of the SAS library to personal specification */

libname perm 'G:\Home\Research\REALISE-Uganda\Estimation';

/* The generation of results for Table 7 require 3 seperate reverse simulations. See Section 5 of the paper for further details. This file is used to produce estimates for

theta13. */

**%macro** ***interim***;

data sim;

set sim;

array n[**16**, **12**];

array s[**16**, **12**];

array z[**24**, **12**];

array v[**24**, **12**];

array invarone[**24**];

array zsum[**6**, **12**];

array vsum[**6**, **12**];

array varonesum[**6**];

array c[**6**, **12**];

array sinput[**16**];

array lastint[**4**];

array treat[**16**];

array conc[**6**];

interim = interim - **1**;

seed = **477568890** + interim;

call streaminit(seed);

do st = **1** to **16**;

if interim < lastint[treat[st]] and s[st, interim + **1**] > **0** then do;

s[st, interim] = rand('HYPER', n[st, interim + **1**], s[st, interim + **1**], n[st, interim]);

end;

end;

do site = **1** to **4**;

do tra = **1** to **3**;

do trb = tra + **1** to **4**;

comp = **6***(site - **1**) + (tra = **1**)*(trb - **1**) + (tra = **2**)*(trb + **1**) + (tra = **3**)*(trb + **2**);

sta = site + **4***(tra - **1**);

stb = site + **4***(trb - **1**);

if interim <= lastint[tra] and interim <= lastint[trb] then do;

z[comp, interim] = ((n[stb, interim]*s[sta, interim] - n[sta, interim]*s[stb, interim])/(n[sta, interim] + n[stb, interim]));

v[comp, interim] = ((n[sta, interim]*n[stb, interim]*(s[sta, interim] + s[stb, interim])

*(n[sta, interim] + n[stb, interim] - s[sta, interim] - s[stb, interim])/((n[sta, interim] + n[stb, interim])****3**)));

end;

if interim = **1** then do;

invarone[comp] = ((n[sta, **1**] + n[stb, **1**])*v[comp, **1**])/(n[sta, **1**] + n[stb, **1**] - **1**);

/**************************************************************

The (-1) in the above line is explained in section 5 of the paper, specifically in equation 7.

**************************************************************/

end;

end;

end;

end;

stillin = **0**;

equiv = **0**;

do tra = **1** to **3**;

do trb = tra + **1** to **4**;

komp = (tra = **1**)*(trb - **1**) + (tra = **2**)*(trb + **1**) + (tra = **3**)*(trb + **2**);

zsum[komp, interim] = z[komp, interim] + z[komp + **6**, interim] + z[komp + **12**, interim] + z[komp + **18**, interim];

vsum[komp, interim] = v[komp, interim] + v[komp + **6**, interim] + v[komp + **12**, interim] + v[komp + **18**, interim];

if interim = **1** then do;

varonesum[komp] = **1**/(invarone[komp] + invarone[komp + **6**] + invarone[komp + **12**] + invarone[komp + **18**]);

/**************************************************************

The (-1) in the above line is explained in section 5 of the paper, specifically in equation 7.

**************************************************************/

end;

b1 = -int - slope*vsum[komp, interim];

b2 = int - **3***slope*vsum[komp, interim];

if (b2 > **0**) then b2 = **0**;

b3 = -b2;

b4 = -b1;

if (b2 > **0**) then b2 = **0.0001**;

if (b3 < **0**) then b3 = **0.0001**;

c[komp, interim] = ((**1** + (zsum[komp, interim] > b1) + (zsum[komp, interim] >= b2) + (zsum[komp, interim] > b3) + (zsum[komp, interim] >= b4)));

if interim = lastint[tra] and interim = lastint[trb] then do;

stillin = stillin + **1**;

end;

if interim = lastint[tra] and interim < lastint[trb] then do;

complete = min(complete, (**1** - (c[komp, interim] = **5**)));

complete = min(complete, (**1** - (c[komp, interim] > **1**)*(conc[komp] = **1**)));

complete = min(complete, (**1** - (c[komp, interim] = **1**)*(conc[komp] > **1**)));

stillin = stillin + **1**;

equiv = equiv + (c[komp, interim] = **3**);

end;

if interim < lastint[tra] and interim = lastint[trb] then do;

complete = min(complete, (**1** - (c[komp, interim] = **1**)));

complete = min(complete, (**1** - (c[komp, interim] < **5**)*(conc[komp] = **5**)));

complete = min(complete, (**1** - (c[komp, interim] = **5**)*(conc[komp] < **5**)));

stillin = stillin + **1**;

equiv = equiv + (c[komp, interim] = **3**);

end;

if interim < lastint[tra] and interim < lastint[trb] then do;

complete = min(complete, **1** - (c[komp, interim] = **1**) - (c[komp, interim] = **5**));

stillin = stillin + **1**;

equiv = equiv + (c[komp, interim] = **3**);

end;

if interim <= lastint[tra] and interim <= lastint[trb] then do;

complete = min(complete, **1** - (equiv = stillin));

end;

end;

end;

run;

proc means data = sim;

var interim;

run;

**%mend**;

**%macro** ***results***;

data results;

set sim;

array z[**24**, **12**];

array v[**24**, **12**];

array zsum[**6**, **12**];

array vsum[**6**, **12**];

array varonesum[**6**];

array thetahat[**6**];

array c[**6**, **12**];

array sinput[**16**];

array lastint[**4**];

array treat[**16**];

array conc[**6**];

if complete = **0** then delete;

do comp = **1** to **6**;

thetahat[comp] = zsum[comp, **1**]*varonesum[comp];

end;

run;

proc means data = results noprint;

var thetahat1 thetahat2 thetahat3 thetahat4 thetahat5 thetahat6 varonesum1 varonesum2 varonesum3 varonesum4 varonesum5 varonesum6;

output out = summary mean(thetahat1) = th12 mean(thetahat2) = th13 mean(thetahat3) = th14

mean(thetahat4) = th23 mean(thetahat5) = th24 mean(thetahat6) = th34

var(thetahat1) = var_th12 var(thetahat2) = var_th13 var(thetahat3) = var_th14

var(thetahat4) = var_th23 var(thetahat5) = var_th24 var(thetahat6) = var_th34

mean(varonesum1) = vee12 mean(varonesum2) = vee13 mean(varonesum3) = vee14

mean(varonesum4) = vee23 mean(varonesum5) = vee24 mean(varonesum6) = vee34;

run;

data summary;

set summary;

array th[**6**] th12 th13 th14 th23 th24 th34;

array var_th[**6**] var_th12 var_th13 var_th14 var_th23 var_th24 var_th34;

array se_th[**6**] se_th12 se_th13 se_th14 se_th23 se_th24 se_th34;

array vee[**6**] vee12 vee13 vee14 vee23 vee24 vee34;

array thl[**6**] thl12 thl13 thl14 thl23 thl24 thl34;

array thu[**6**] thu12 thu13 thu14 thu23 thu24 thu34;

do comp = **1** to **6**;

se_th[comp] = sqrt(vee[comp] - var_th[comp]);

thl[comp] = th[comp] - **1.96***se_th[comp];

thu[comp] = th[comp] + **1.96***se_th[comp];

end;

run;

proc print data = summary;

run;

**%mend**;

**%macro** ***evaluate***;

%do u = **1** %to **12**;

%***interim***;

%end;

%***results***;

**%mend**;

**data** input;

array n[**16**, **12**];

array sinput[**16**];

array lastint[**4**];

array conc[**6**];

input n1-n192 sinput1-sinput16 lastint1-lastint4 conc1-conc6;

cards;

11 18 30 41 50 57 65 76 86 92 98 103

10 16 25 33 41 49 60 71 82 88 96 100

7 17 25 35 44 55 63 68 72 83 90 104

8 21 28 35 45 55 64 73 84 97 112 125

12 24 31 39 0 0 0 0 0 0 0 0

6 13 25 30 0 0 0 0 0 0 0 0

7 16 22 35 0 0 0 0 0 0 0 0

11 19 30 40 0 0 0 0 0 0 0 0

9 19 29 39 48 57 67 74 85 91 102 111

7 15 24 32 40 49 57 64 72 79 88 94

9 17 25 32 42 50 58 68 76 90 101 111

11 21 30 41 50 60 70 82 91 100 105 116

9 15 23 36 50 0 0 0 0 0 0 0

9 20 32 42 47 0 0 0 0 0 0 0

11 19 28 32 40 0 0 0 0 0 0 0

7 18 25 34 43 0 0 0 0 0 0 0

83 67 64 68 25 13 21 11 85 56 60 45 32 27 18 16

12 4 12 5

5 5 5 0 0 0

;

**run**;

**data** sim;

set input;

array n[**16**, **12**];

array s[**16**, **12**];

array z[**24**, **12**];

array v[**24**, **12**];

array zsum[**6**, **12**];

array vsum[**6**, **12**];

array c[**6**, **12**];

array sinput[**16**];

array lastint[**4**];

array treat[**16**];

array conc[**6**];

int = **10.90266**;

slope = **0.12380**;

interim = **13**;

complete = **1**;

do i = **1** to **12**;

do st = **1** to **16**;

s[st, i] = **0**;

end;

end;

do i = **1** to **12**;

do comp = **1** to **24**;

z[comp, i] = **0**;

v[comp, i] = **0**;

end;

end;

do i = **1** to **12**;

do komp = **1** to **6**;

zsum[komp, i] = **0**;

vsum[komp, i] = **0**;

c[komp, i] = **0**;

end;

end;

do st = **1** to **16**;

treat[st] = int((st + **3**)/**4**);

s[st, lastint[treat[st]]] = sinput[st];

end;

/*******************************************************************

Here is where the number of reverse simulations is set. We have used 10,000,000 for Table 7.

*******************************************************************/

do rep = **1** to **10000000**;

output;

end;

**run**;

%***evaluate***;
